# Egg Albumin-Assisted Hydrothermal Synthesis of Co_3_O_4_ Quasi-Cubes as Superior Electrode Material for Supercapacitors with Excellent Performances

**DOI:** 10.1186/s11671-019-3172-y

**Published:** 2019-11-11

**Authors:** Jiale Sun, Ya Wang, Yanfei Zhang, Chunju Xu, Huiyu Chen

**Affiliations:** School of Materials Science and Engineering, North University of China, Taiyuan, 030051 China

**Keywords:** Co_3_O_4_, Hydrothermal synthesis, Electrochemical properties, Supercapacitors

## Abstract

Novel Co_3_O_4_ quasi-cubes with layered structure were obtained via two-step synthetic procedures. The precursors were initially prepared via hydrothermal reaction in the presence of egg albumin, and then the precursors were directly annealed at 300 °C in air to be converted into pure Co_3_O_4_ powders. It was found that the size and morphology of final Co_3_O_4_ products were greatly influenced by the amount of egg albumin and hydrothermal durations, respectively. Such layered Co_3_O_4_ cubes possessed a mesoporous nature with a mean pore size of 5.58 nm and total specific surface area of 80.3 m^2^/g. A three-electrode system and 2 M of KOH aqueous electrolyte were employed to evaluate the electrochemical properties of these Co_3_O_4_ cubes. The results indicated that a specific capacitance of 754 F g^−1^ at 1 A g^−1^ was achieved. In addition, the Co_3_O_4_ cubes-modified electrode exhibited an excellent rate performance of 77% at 10 A g^−1^ and superior cycling durability with 86.7% capacitance retention during 4000 repeated charge-discharge process at 5 A g^−1^. Such high electrochemical performances suggest that these mesoporous Co_3_O_4_ quasi-cubes can serve as an important electrode material for the next-generation advanced supercapacitors in the future.

## Introduction

With the fast development of science and technology in modern society, relying solely on fossil fuels with limited storage is far from meeting the ever-increasing requirements of energy, so some new energy storage devices with environmental-benign types have been developed rapidly to solve this dilemma [1–3]. At present, batteries and supercapacitors are two types of the most promising energy storage systems because of their high performance and low cost. In particular, supercapacitors, also known as electrochemical capacitors, have attracted more attention in terms of their excellence in power density, long-term cycling life, charge-discharge rate, and other properties [4–6]. Attributed to such advantages, supercapacitors have been applied in emergency lighting, hybrid electric vehicles, military equipment, and short-term power sources [7, 8]. At the same time, the energy and power density of supercapacitors need to be continuously increased to accommodate the expansion of their application fields; as a result, tremendous efforts have been devoted to resolving this problem. Achieving noteworthy improvements in supercapacitors require a deep fundamental understanding of the charge storage mechanisms. It has been found that the shape, porosity, as well as mechanical properties of electrode materials have a crucial impact on the performances of supercapacitors [9–11]. For an ideal electrode material, the number of electrochemically active sites for charge transfer should be enhanced and ionic/electronic transport should be controlled at small diffusion length [12].

Supercapacitors have been differed on the categories on the basis of different energy storage mechanisms. One of them stores energy by charge accumulation at the interface of electrode and electrolyte, and it is known as electric double-layer capacitors (EDLCs). The other is pseudo-capacitors (PCs), which rely on fast Faradic reaction occurring near/on the surface of electrode materials to store energy [13–16]. The carbonaceous materials, such as activated carbon, graphene, and carbon nanotubes (CNTs) that have large specific surface area and good conductivity, are ideal electrode materials for EDLCs. However, for the carbon-based materials, their inherently low specific capacitance is a sever defect that cannot be ignored, which leads to lower energy density than that of PCs [17]. Conductive polymers as well as metal oxides are commonly used as electrode materials in PCs, due to their favorable pseudocapacitive characteristics of fast and reversible redox reactions. PCs can provide higher energy and power density, larger specific capacitance, and have attracted worldwide research interest [18]. To date, metal oxides, especially transition metal oxides (TMOs), such as MnO_2_ [19, 20], NiO [21, 22], and Fe_2_O_3_ [23, 24], have attracted much attention as potential candidate for electrode materials, for they can provide rich redox charge transfer originated from their variety of oxidation states, which is beneficial to the Faraday reaction. Despite the virtues of low cost and high specific capacitance, the effects of these materials used as electrode in PCs are still not satisfactory, given the fact that they generally possess dramatic volume change, inferior rate-capability, and relatively high resistance; enormous efforts have been devoted to circumvent the hurdles [25]. Among the series of TMOs, Co_3_O_4_ is considered as one of the most promising electrode materials. This kind of material possesses a theoretical specific capacitance as high as 3560 F g^−1^ [26]. Besides, it is environmentally friendly, cheap, and rich in redox activity as well. Unfortunately, compared to its theoretical value, the specific capacitance of Co_3_O_4_ electrode achieves in practical applications is significantly low. Ascribed to the limitation transfer of electrons caused by the high internal resistance of Co_3_O_4_, only a part of active sites may be involved in the redox reaction, leading to low utilization of the active material and decrease in specific capacitance. Furthermore, the Co_3_O_4_ has a dramatic volume change trend during the process of rapid redox reactions, and the collapse of the electrode material leads to a reduction of the cycle life [27].

To address these problems, Co_3_O_4_ nanostructures with different morphologies, including nanorods, nanowires, nanoflakes, and nanoflowers, have been successfully prepared by controlling the synthesis process, aiming to increase the surface area and facilitate the redox reaction [28–31]. The research results have shown that different morphologies have a significant effect on the performance of Co_3_O_4_ electrode, but merely changing the morphology is far from being able to improve its inherent poor conductivity and serious volume expansion defects. Researchers are devoted to combine Co_3_O_4_ with other highly conductive materials to obtain electrode materials with high charge transfer capabilities. In addition, the synergy between different materials can contribute to the redox reaction at the same time, to achieve the purpose of increasing the specific capacitance [32–35]. From the point of practical applications and large production, it is significantly important to prepare powder electrode material through a simple synthetic process.

Solution method including hydrothermal/solvothermal route is one of the important synthetic strategies to prepare micro/nanomaterials on a large scale. In this method, surfactant is usually employed to control the rate of nucleation and crystal growth. So the final shape of nanostructures can be effectively tuned by the surfactant [36–38]. Several types of surfactant including cationic surfactant, anionic surfactant, nonionic surfactant, and so on can be used for the fabrication of nanomaterials. Among them, the biological molecules with functional groups have received increasing attention due to the environmental-benign of this kind of surfactant. The proteins can interact with inorganic nanoparticles and then to govern the nucleation of inorganic materials in aqueous solutions. Egg albumin, as an important protein, can be widely available from eggs. It has received much attention due to its gelling, foaming, and emulsifying characteristics. In addition, egg albumin is cost-effective and environmentally friendly, and the usage of such surfactant may not result in danger for both environment and the health of humans. Therefore, egg albumin can be employed for the preparation of nanomaterials with controlled morphology. For example, Geng et al. prepared single crystalline Fe_3_O_4_ nanotubes with high yields using egg albumin as a nanoreactor [39]. ZnS nanosheets can be synthesized via egg albumin and microwave-assisted method [40]. In addition, dumbbell-shaped BaCO_3_ superstructures and SnO_2_ biscuits can be obtained with the assistance of egg albumin by different research group [41, 42]. Overall, the reports on nanomaterials fabrication involving egg albumin have been rarely reported. In this work, porous Co_3_O_4_ cubes were synthesized with the assistance of egg albumin via a hydrothermal method and post calcination of the precursors. These Co_3_O_4_ porous cubes had average pore size of 5.58 nm, and the Brunauer-Emmett-Teller (BET) specific surface area was evaluated to be 80.3 m^2^/g. If such Co_3_O_4_ cubes were processed into a working electrode, a high capacitance of 754 F g^−1^ was obtained at 1 A g^−1^. Besides, if the current density was improved to 10 A g^−1^, the electrode showed a high rate capability up to 77%. A superior cycling performance with 86.7% capacitance retention (at 5 A g^−1^) was also achieved during 4000-cycle charge-discharge process. Such excellent electrochemical properties indicate that the porous Co_3_O_4_ cubes can serve as a promising electrode material for supercapacitors in the near future.

## Methods

### Materials

In this work, all reagents were in analytical pure grade and were used without any additional purification. Urea and cobalt (II) acetate tetrahydrate were purchased from Sinopharm Chemical Reagent Co., Ltd., and egg albumin was obtained from fresh eggs.

### Preparation of Porous Co_3_O_4_ Cubes

To prepare the porous Co_3_O_4_ cubes, 3 mL of egg albumin, 2.4 g of urea, and 0.3 g of cobalt (II) acetate tetrahydrate were dissolved in 37 mL of de-ionized (DI) water with vigorous stirring. Then the mixture was loaded into an autoclave with 50 mL of capacity, and the autoclave was placed in an oven at 140 °C. Five hours later, the precipitates were harvested, rinsed, and dried at 60 °C overnight. The obtained precursor was annealed at 300 °C for 5 h in order that black powder was obtained. Control experiments were conducted with various hydrothermal time (1, 2, 15, and 24 h) and different amount of egg albumin, respectively, while keeping other parameters and procedures the same.

### Fabrication of Working Electrode and Electrochemical Tests

On a CHI 660E electrochemical workstation, three kinds of electrochemical tests including cyclic voltammetry (CV), chronopotentiometry (CP), and electrochemical impedance spectroscopy (EIS) were performed based on typical three-electrode configuration, in which platinum wire served as counter electrode and saturated calomel electrode (SCE) was used as reference electrode, respectively. Detailed description about the fabrication of working electrode was as follows: a mixed powder containing active material, acetylene black, and polyvinylidene fluoride (PVDF) with weight ratio of 80:15:5 was prepared firstly, and then the mixed powder was dispersed in *N*-methyl-2-pyrrolidone (NMP) solvent under ultrasound assistance. The obtained suspension was coated onto pre-cleaned nickel foam (1 × 1 cm^2^) and vacuum-dried at 85 °C; subsequently, a pressure of 10 MPa supplied by hydraulic press was performed on the nickel foam and the working electrode was finally obtained. All the tests were carried out in 2 M of KOH aqueous electrolyte; the potential of CV tests varied from − 0.1 to 0.65 V, and the scan rates were ranging in 2–50 mV s^−1^. For the CP tests, the current density differed from 1 to 10 A g^−1^ with the potential varying from 0 to 0.45 V. An open circuit potential was adopted for the EIS measurement; the frequency region was 10^−2^–10^5^ Hz and the AC amplitude was 5 mV. The specific capacitance can be obtained from Eq. (1):

1 $$ {C}_s=\frac{I\cdot \Delta t}{m\cdot \Delta V} $$

where *C*_*s*_ (F g^−1^) represents the specific capacitance, ∆*t* (s) indicates discharging time, *I* (A) is discharging current, ∆*V* (V) means potential window, and *m* (g) is the weight of active material.

## Characterizations

The X-ray diffraction (XRD) pattern of the sample was collected on a powder X-ray diffractometer (Bruker D8 Advance), in which Cu-kα was used as X-ray source (λ = 0.1548 nm) and the range of 2θ was 25–100°. Field-emission electron microscope (FESEM) images were available from a JEOL JSM7100F scanning electron microscope, and transmission electron microscope (TEM) image was obtained on JEOL JEM2100F equipment with operation voltage of 200 kV. Before TEM measurement, the powder needs to be ultrasonically dispersed in ethanol for 10 min, then is dropped onto a carbon-coated copper grid. Raman examination was performed on RM 1000-Invia (Renishaw) spectrometer, and the wavelength for laser was chosen to be 514 nm. X-ray photoelectron spectroscopy (XPS) measurement was operated on ESCA 2000 spectrometer and Al Kα was employed as excitation source. According to nitrogen adsorption/desorption experiments conducted at 77 K, the Brunauer-Emmet-Teller (BET) surface area was obtained. In addition, pore size distribution (Barrett-Joyner-Halenda, BJH method) could be acquired from the related desorption isotherm.

## Results

**Fig. 1:**
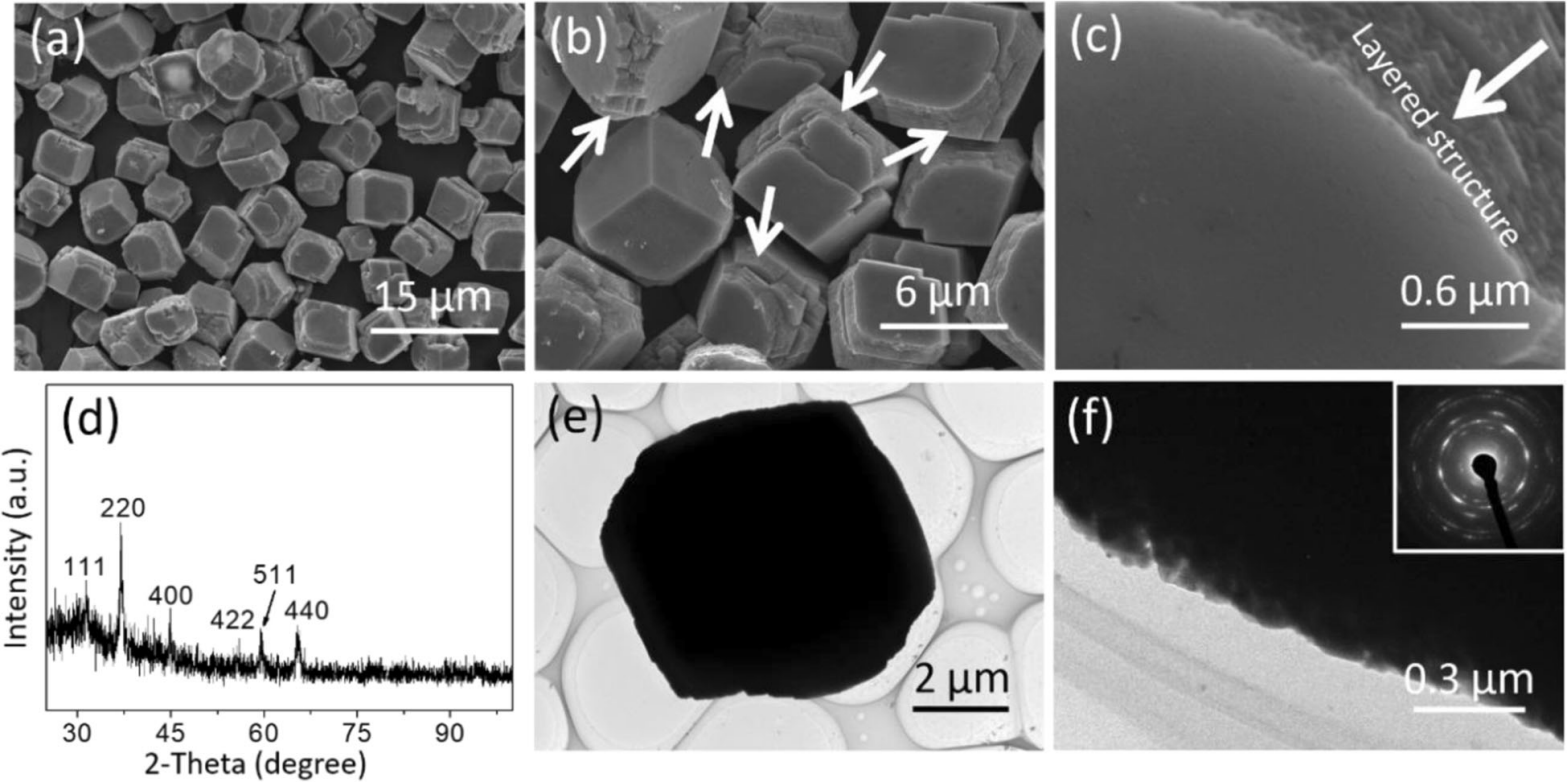
**a–c** SEM images, **d** XRD pattern, and **e**, **f** TEM images of porous Co_3_O_4_ quasi-cubes with the SAED pattern in the inset of (**f**)

The shape and size of the sample prepared with 3 mL of egg albumin at 140 °C for 5 h combined with a post annealing process at 300 °C were investigated by SEM (Fig. 1a). It indicated that the product was dominated by a huge amount of cube-like particles with size of about 5–6 μm. The enlarged SEM image (Fig. 1b) demonstrated that some corners for each cube were not perfect, and the cube was assembled with layered structures, as the white arrows pointed. Such novel layered structure could be clearly observed from the SEM image in Fig. 1c. The composition and crystal phase were investigated by XRD technique. Figure 1d displayed the typical XRD pattern, in which all the observed diffraction peaks can be indexed as (111), (220), (400), (422), (511), and (440) crystal planes of cubic Co_3_O_4_ (JCPDS No. 43-1003). There were no peaks generated from impurities of Co(OH)_2_ and CoO, suggesting high purity of the Co_3_O_4_ sample obtained herein. The TEM image in Fig. 1e showed an representative Co_3_O_4_ cube with size of 5 μm, and the size was in good agreement with the SEM data. Figure 1f exhibited a magnified TEM image that was taken from the position focused on an edge of the cube. The porous structure could be seen, so the total Co_3_O_4_ cube was actually composed of many nanoparticle (NP)-based layers. The selected area electron diffraction (SAED) pattern indicated polycrystalline structure, and the spot-based diffraction rings further suggested the large amount of assembled NPs in the porous Co_3_O_4_ cube. In addition, the cube was so thick that high-resolution TEM (HRTEM) characterization was difficult to carry out.

**Fig. 2:**
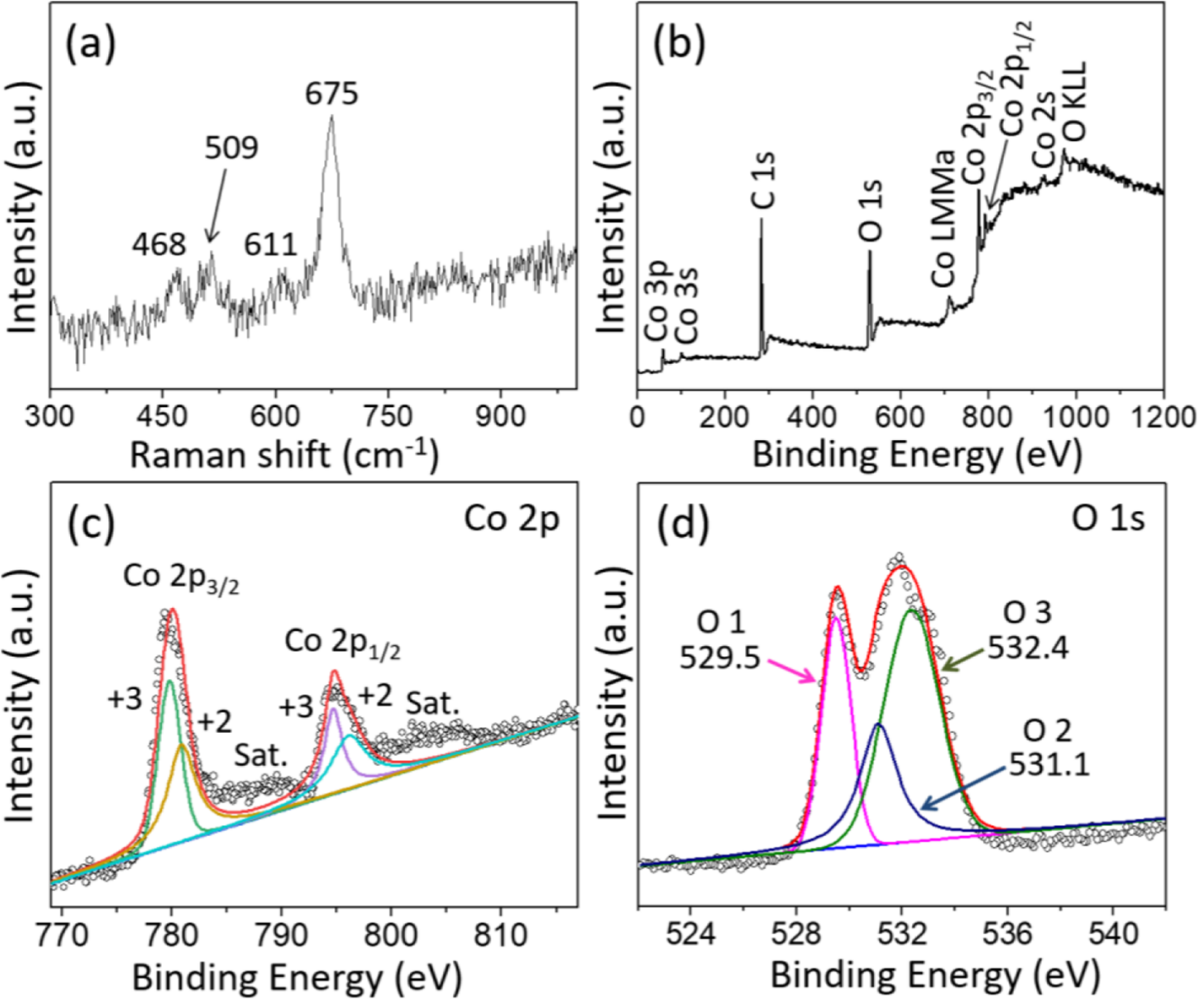
**a** Raman spectrum and **b** XPS survey spectrum of porous Co_3_O_4_ cubes, and the core-level spectra for **c** Co 2p and **d** O 1s

The detailed structural information of Co_3_O_4_ nanocubes was evaluated by Raman spectrum shown in Fig. 2a. Four characteristic bands located at 468, 509, 611, and 675 cm^−1^ can be observed, which were corresponding to E_g_, ${\mathrm{F}}_{2\mathrm{g}}^1$, ${\mathrm{F}}_{2\mathrm{g}}^2$, and A_1g_ Raman-active modes, respectively. Such results were in good agreement with previous literatures, further demonstrating the formation of Co_3_O_4_ [33, 43]. Figure 2b illustrated a representative full survey XPS spectrum of Co_3_O_4_ nanocubes, and no other peaks of impurity can be observed except for the characteristic peaks of carbon, cobalt, and oxygen elements. The full survey XPS data suggested the high purity of the Co_3_O_4_ sample. As can be seen from the high resolution of Co 2p spectrum exhibited in Fig. 2c, there are two obvious peaks centered at 779.7 and 794.8 eV, which are corresponding to Co 2p_3/2_ and Co 2p_1/2_, respectively. In addition, the energy separation of the two peaks was 15.1 eV, reflecting the existence of Co^3+^ [14]. Moreover, the two main peaks could be separated into two spin-orbit doublets after Gaussian fitting; the peaks with 779.6 and 794.6 eV binding energies were related to Co^3+^, whereas others located at 780.9 as well as 796.2 eV were corresponding to Co^2+^; such observation results matched well with previous report [44]. Two satellite peaks (marked as “sat”) can be observed near the binding energies of 788.6 and 804.1 eV, and their presence further confirmed the characteristic of spinel structures [45]. The fitting results of O 1s spectrum (Fig. 2d) displayed that there were three oxygen contributions (O1, O2, O3). The O1 component located at 529.5 eV can be indexed to typical metal-oxygen bonding, and the O2 component at 531.1 eV is ascribed to the hydroxyl group [46]. As for O3 component with high binding energy of 532.4 eV, it is corresponding to the water molecules absorbed on the electrode surface [47].

**Fig. 3:**
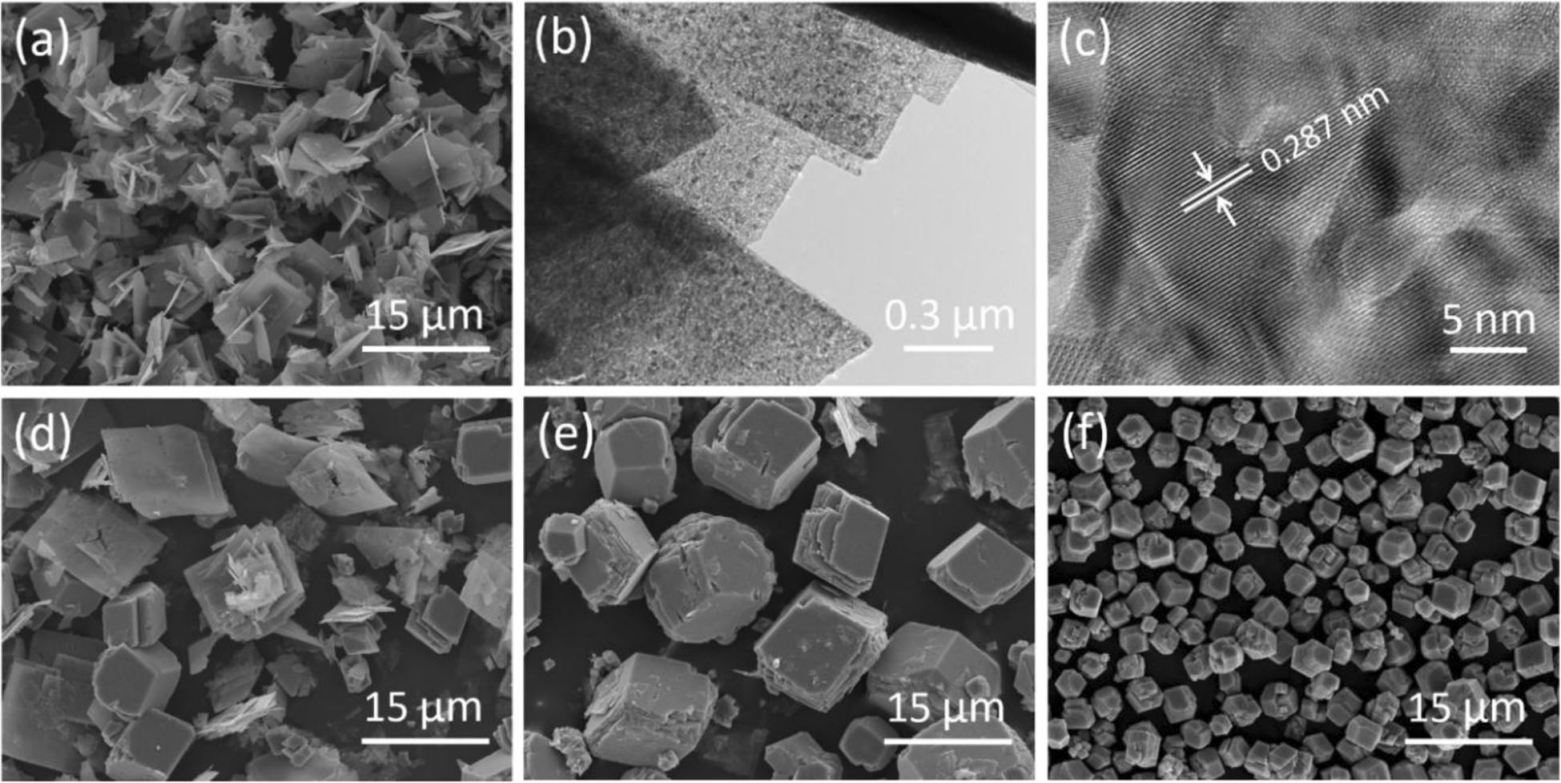
**a** SEM image and **b**, **c** TEM images of Co_3_O_4_ nanosheets obtained without any egg albumin, and the SEM images of the Co_3_O_4_ samples prepared with egg albumin of **d** 0.5, **e** 1, and **f** 5 mL

The amount of egg albumin in the system played a key role to form such Co_3_O_4_ cubes. If no egg albumin was employed, the product was dominated by a lot of Co_3_O_4_ nanosheets (Fig. 3a), and the porous structure could be clearly found in the TEM image in Fig. 3b. Such Co_3_O_4_ nanosheets were well crystallined; besides, the 0.287 nm of fringe spacing (Fig. 3c) corresponded to the (220) crystal planes of Co_3_O_4_. When 0.5 mL of egg albumin was added, the Co_3_O_4_ sample was composed of some layered cubes as well as some sheets (Fig. 3d). Co_3_O_4_ nanosheets almost completely disappeared as the dosage of egg albumin was increased to 1 mL. Under this condition, Co_3_O_4_ qusi-cubes with edge length of about 3–10 μm were formed (Fig. 3e). Uniformed Co_3_O_4_ cubes could be obtained as the amount of egg albumin was continuously increased to 3 mL. If we further increased the egg albumin to 5 mL, the morphology of the Co_3_O_4_ cubes were well preserved without any change, but the size was reduced to 3–4 μm (Fig. 3f). From the above SEM observations, the formation process of Co_3_O_4_ cubes with the assistance of egg albumin can be tentatively proposed. During the reaction, Co^2+^ ions react with egg albumin to form a complex; the combination of nitrogen-atoms in the egg albumin molecules and Co^2+^ ions can promote the aggregate growth. Due to the stacking interactions and crystal packing force, the aggregates prefer to grow into flake structure. If the amount of egg albumin is sufficient enough, the flakes tend to be stacked owing to the existence of hydrogen bonds among the molecules, leading to the formation of final layered cube structures.

**Fig. 4:**
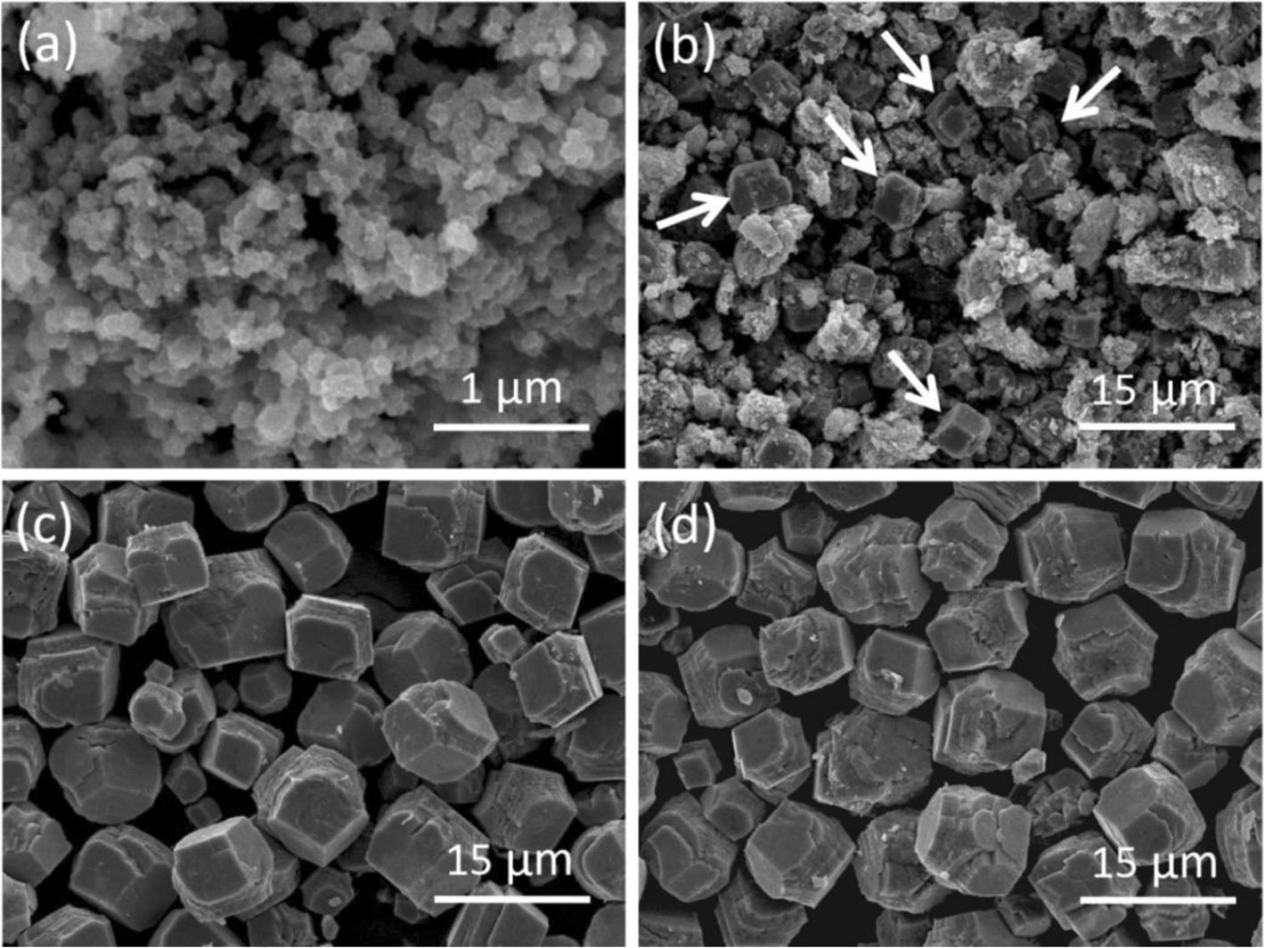
SEM images of the Co_3_O_4_ samples synthesized with different hydrothermal durations: **a** 1, **b** 2, **c** 15, and **d** 24 h

Controlled experiments were also conducted with different hydrothermal reaction time while the dosage of egg albumin was fixed at 3 mL. If the reaction proceeded only 1 h, Co_3_O_4_ NPs with irregular shapes were produced in large quantities (Fig. 4a). A small amount of Co_3_O_4_ cubes and NPs coexisted when the reaction was extended to 2 h (Fig. 4b). Perfect Co_3_O_4_ cubes could be obtained on a large scale as the hydrothermal treatment was prolonged to 5 h; after that, the shape and size almost had no obvious chage with the reaction prolonging to 15 h or longer (Fig. 4c, d). The growth mechanism of Co_3_O_4_ cubes and the influence of egg albumin on the final Co_3_O_4_ morphology require further detailed investigations, and related research is currently ongoing.

**Fig. 5:**
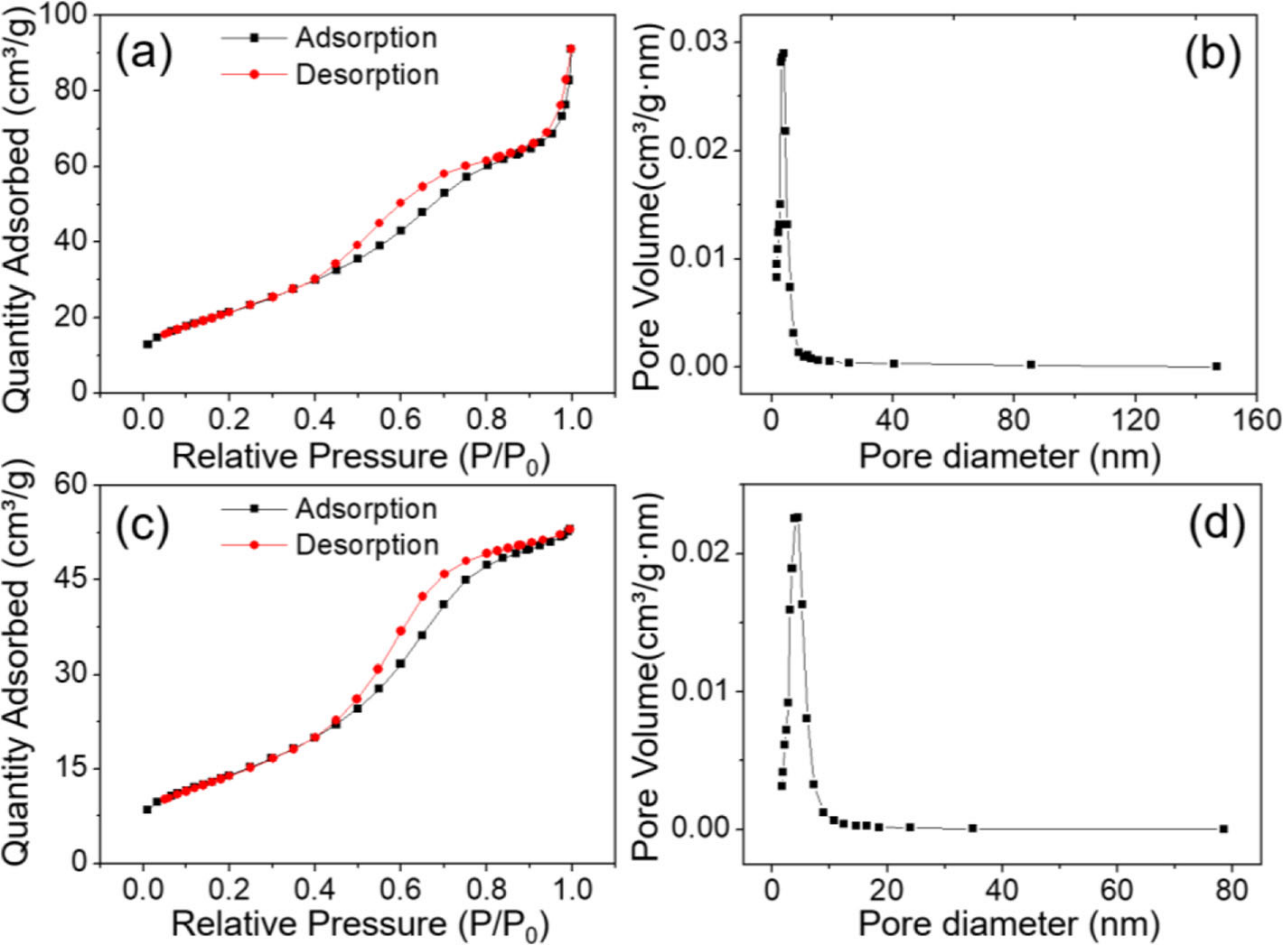
N_2_ adsorption-desorption isotherms and the corresponding BJH pore size distributions for **a**, **b** porous Co_3_O_4_ cubes and **c**, **d** porous Co_3_O_4_ nanosheets, respectively

The porosity of these Co_3_O_4_ cubes was investigated by nitrogen adsorption-desorption isotherms. The mesoporous structure properties of Co_3_O_4_ nanocubes could be clearly revealed by the isotherms shown in Fig. 5a, for such isotherms were categorized as a typical IV type and accompanied with a H3-type hysteresis. The pore size distribution obtained by BJH method further proved this point (Fig. 5b). The average pore diameter of these Co_3_O_4_ nanocubes was 5.58 nm, and the BET specific surface area was evaluated to be 80.3 m^2^ g^−1^. Especially, it was seen from Fig. 5b that the pore size was dominantly distributed over 4.03 nm. The isotherms of Co_3_O_4_ nanosheets was illustrated Fig. 5c, which were similar to the isotherms of nanocubes; however, the BET-specfic surface area of Co_3_O_4_ nanosheets was lower than Co_3_O_4_ nanocubes, only 52.5 m^2^ g^−1^. In addition, the average pore diameter of Co_3_O_4_ nanosheets aquaired from Fig. 5d was 4.44 nm. It is well known that electrode materials with large surface area and porosity are more favorable for rapid electrochemical reactions in that the number of electrochemically active sites increases, and the transport of electrons as well as ions accelerates. Attribute to well-distributed pore diameter and large surface area, the Co_3_O_4_ nanocubes-modified electrode enable to provide rapid progress of redox reaction and easy penetration of the electrolyte within the electrode, leading to a favorable specific capacitance.

**Fig. 6:**
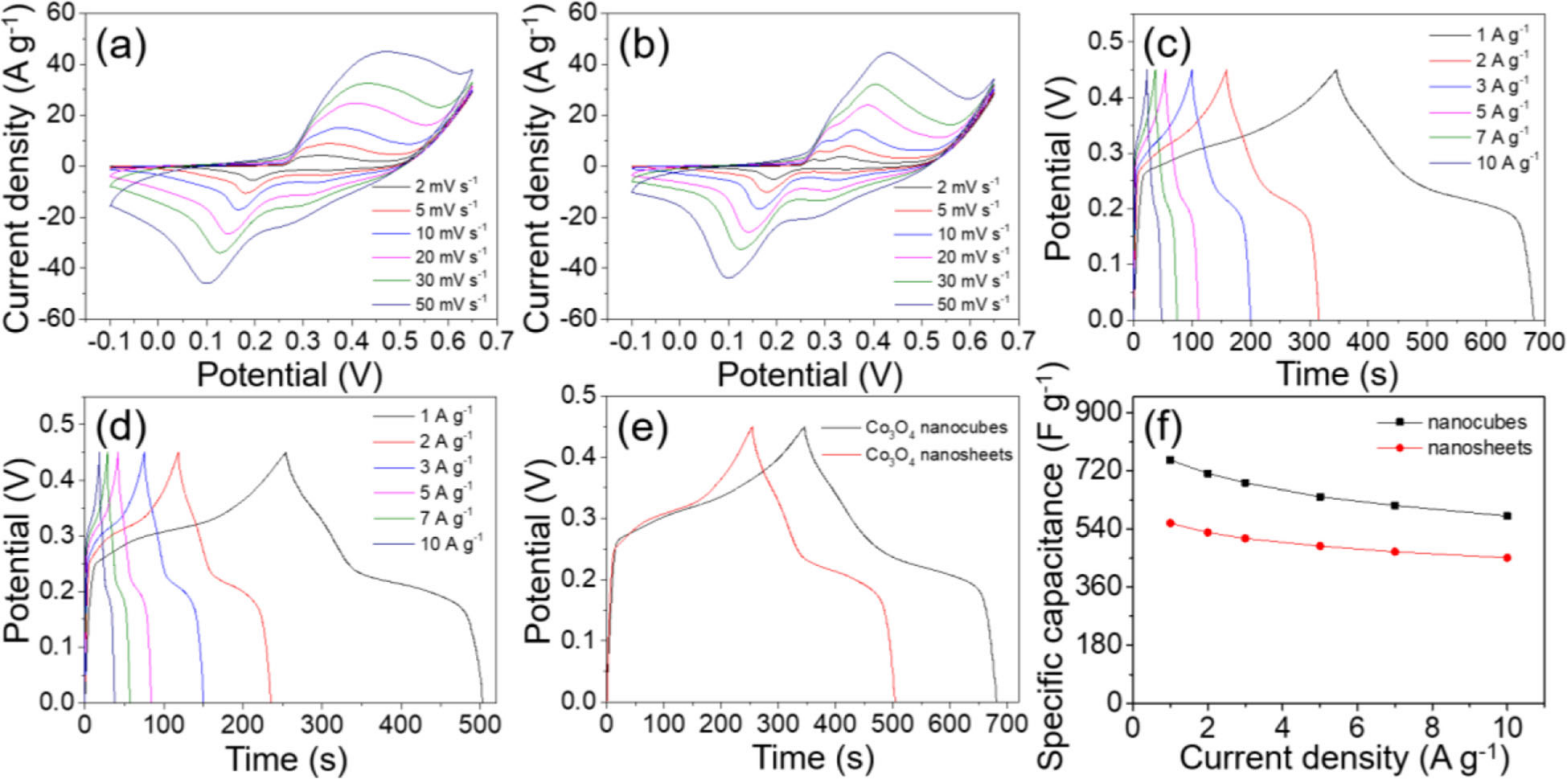
CV curves measured with different scan rates for **a** porous Co_3_O_4_ cubes and **b** porous Co_3_O_4_ nanosheets, CP curves measured with different current densities for **c** porous Co_3_O_4_ cubes and **d** porous Co_3_O_4_ nanosheets, **e** CP curves of the two electrodes obtained at 1 A g^−1^, and **f** specific capacitances obtained at various current densities

The electrochemical performance of as-prepared Co_3_O_4_ nanocubes was evaluated by CV, CP, and EIS measurements. All the tests were conducted in 2 M of KOH aqueous electrolye using a three-electrode configuration. With the potential varying from − 0.1 to 0.65 V and the scan rate shifting between 2 and 50 mV s^−1^, the CV curves of Co_3_O_4_ nanocubes and nanosheets were presented in Fig. 6a, b, respectively. Both the CV curves had more than one pair of well-defined reduction and oxidation peaks. Such phenomenon implied that the charge storage for Co_3_O_4_ nanocubes electrode was governed by pseudocapacitance instead of electrical double-layer capacitance that exhibits rectangular CV curves [48]. Based on the difference in morphology and porosity, the CV curves of the two electrode materials are not completely similar. In terms of the area integrated by CV curves, the Co_3_O_4_ nanocubes-modified electrode is significantly larger than the nanosheets-modified electrode, indicating that the Co_3_O_4_ nanocubes-modified electrode can deliver higher specific capacitance. As Fig. 6a illustrated that the scan rate accelerated, the two oxidation peaks gradually mingled together to form one broad oxidation peak. In addition, the anodic peaks shifted toward more positive position, whereas the reduction peaks moved to more negative position, suggesting the reversible characteristics of the redox reactions [29]. The pairs of redox peaks on both CV curves were corresponding to the conversion among diverse cobalt oxidation states, and the equations were mainly summarized as follows [49]:

2 $$ {\mathrm{Co}}_3{\mathrm{O}}_4+{\mathrm{H}}_2\mathrm{O}+{\mathrm{O}\mathrm{H}}^{-}\leftrightarrow 3\mathrm{CoOOH}+{\mathrm{e}}^{-} $$

3 $$ \mathrm{CoOOH}+{\mathrm{OH}}^{-}\leftrightarrow {\mathrm{CoO}}_2+{\mathrm{H}}_2\mathrm{O}+{\mathrm{e}}^{-} $$

The electrochemical capacitive behaviors of the Co_3_O_4_ nanomaterials were also investigated by CP tests. Figure 6c, d exhibited the CP curves of Co_3_O_4_ nanocubes and nanosheets at various current density, which were acquired over a potential from 0 to 0.45 V. The appearance of distinct potential plateaus in all curves of the two sample demonstrated the pseudocapacitance characteristics, which was consistent with the conclusions obtained from CV curves [50]. According to Eq. (1), the Co_3_O_4_ nanocubes-modified electrode delivered specific capacitances of 754, 712, 683, 641, 614, and 581 F g^−1^, respectively, at the current densities of 1, 2, 3, 5, 7, 10 A g^−1^. As for the Co_3_O_4_ nanosheets-modified electrode, it delivered the specific capacitances of 559, 530, 512, 487, 470, and 452 F g^−1^ at the same test condition. According to the CP curves of the two kinds of electrodes at 1 A g^−1^ (Fig. 6e), it is seen that the discharge time of Co_3_O_4_ cubes-modified electrode is longer than that of the Co_3_O_4_ nanosheets-modified electrode, further demonstrating that the Co_3_O_4_ cubes-modified electrode can display superior electrochemical properties. Figure 6f indicates the variation of specific capacitance at different current density for the two kinds of electrodes. Obviously, the specific capacitance reduces gradually as the current density increases. The rate capabilities of the Co_3_O_4_ nanocubes and nanosheets-modified electrodes from 1 to 10 A g^−1^ were 77% and 81%, respectively. It is not difficult to understand that at high current densities, insufficient diffusion of ions and eletrons makes impossible for electrolyte to achieve full contact with the electrode material, resulting in that only the active sites at the outer surface of the electrode material can participate in the redox reaction. Consequently, the incomplete utilization of the active material directly leads to a reduction in specific capacitance. Compared with other previous related literatures, the Co_3_O_4_ nanocubes-modified electrode synthesized in this work exhibits superior electrochemical performance (Table 1). It is worth mentioning that the composite electrodes formed by the combination of Co_3_O_4_ and other materials tend to exhibit better electrochemical performance. The improved conductivity of composite electrode and the synergy between different substances make a greater contribution to the pesudocapacitance.

**Table 1:** Comparison for the specific capacitances of Co_3_O_4_-based electrode materials

Morphology	BET specific surface area (m^2^/g)	Specific capacitance (F/g)	Ref.
Co_3_O_4_ hollow boxes	31.07	278 F g^−1^ @ 0.5 A g^−1^	[10]
flower-like ZnO/Co_3_O_4_ nanobundle arrays	37	1983 F g^−1^ @ 2 A g^−1^	[18]
mesoporous Co_3_O_4_ nanoflake arrays on carbon cloth	/	450 F g^−1^ @ 1 A g^−1^	[30]
Hierarchical Co_3_O_4_ nanoflowers	49.1	198 F g^−1^ @ 1 A g^−1^	[31]
3D-nanonet hollow structured Co_3_O_4_	92.8	739 F g^−1^ @ 1 A g^−1^	[48]
Co_3_O_4_ nanorods	/	352 F g^−1^ @ 1 A g^−1^	[51]
Flower-like Co_3_O_4_ microspheres	149	214 F g^−1^ @ 2 A g^−1^	[52]
Porous Co_3_O_4_ microspheres	78.6	342.1 F g^−1^ @ 0.5 A g^−1^	[53]
Hollow Co_3_O_4_ nanowire arrays	78	599 F g^−1^ @ 2 A g^−1^	[54]
Hollow fluffy Co_3_O_4_ cages	245.5	948.9 F g^−1^ @ 1 A g^−1^	[55]
Co_3_O_4_ hierarchical micro-and nanostructures	20.5	332.6 F g^−1^ @ 2 mA cm^−2^	[56]
3D Co_3_O_4_@MnO_2_ hierarchical nanoneedle arrays	/	1693.2 F g^−1^ @ 1 A g^−1^	[57]
Co_3_O_4_@highly ordered microporous carbon	/	1307 F g^−1^ @ 1 A g^−1^	[58]
Hierarchical Mo-decorated Co_3_O_4_ nanowire arrays	/	~ 2000 F g^−1^ @ 10 A g^−1^	[59]
Co_3_O_4_ nanocubes	80.3	754 F g^−1^ @ 1 A g^−1^	This work

“/” denotes no related data

**Fig. 7:**
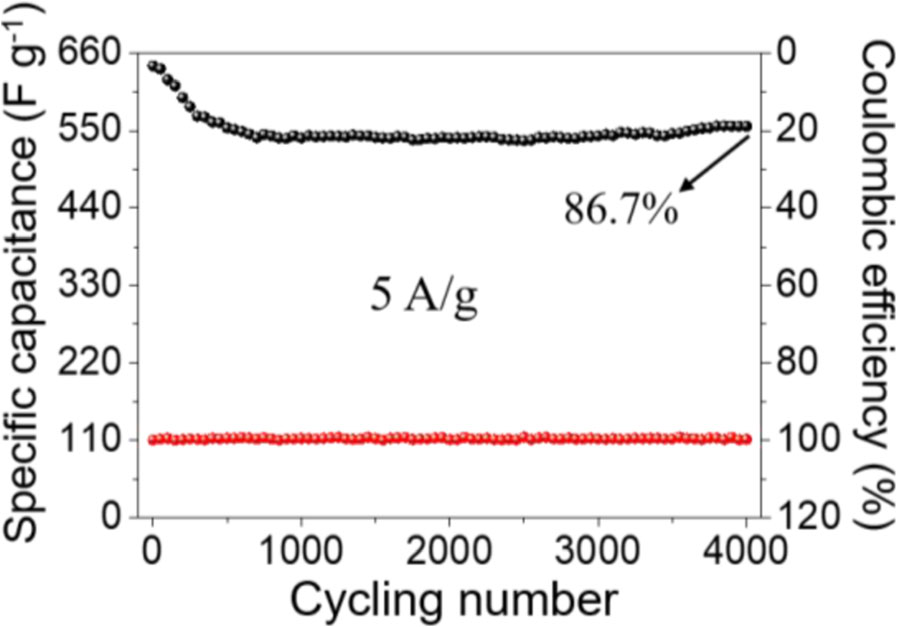
Cyclic stability and Coulombic efficiency of the porous Co_3_O_4_ cubes electrodes measured at 5 A g^−1^

The cycling stability is another vital parameter to measure the appliaction potential of the Co_3_O_4_ nanocubes-modified electrode, which is evaluted by 4000 contious CP tests at 5 A g^−1^. Figure 7 demonstrates that the specific capacitance tends to gradually decrease in the first few hundred cycles, and then stays stable while the cycle number increases; at the end of 4000 cycles, the specific capacitance is 556 F g^−1^ and remains about 86.7% of the initial value. Such results indicate that the Co_3_O_4_ nanocubes-modified electrode possesses excellent long-life cycling durability, which is an important guarantee in supercapacitor applications. The Coulombic efficiency is a parameter that can reflect the reversibility of the redox reaction, which can be calculated by the following equation:

4 $$ \eta =\frac{t_d}{t_c}\times 100\% $$

where *η* represents Coulombic efficiency, *t*_*d*_ and *t*_*c*_ indicate discharge and charge time, respectively. The Coulombic efficiency of Co_3_O_4_ nanocubes-modified electrode almost remains 100% during the entire cycle test (Fig. 7), and it suggests that the pseudocapacitive reactions are remarkably reversible.

**Fig. 8:**
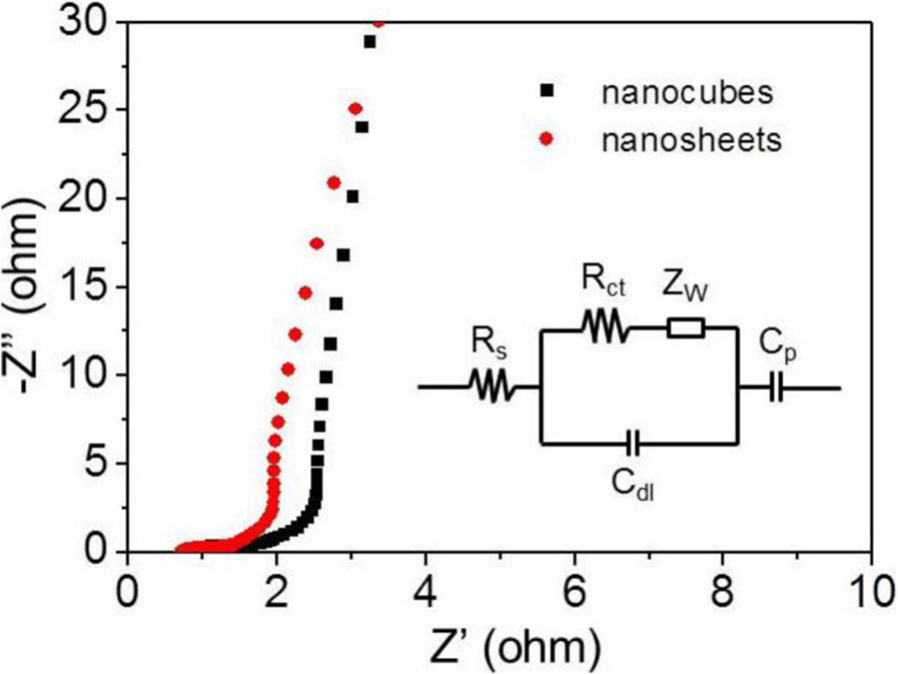
Nyquist plots of Co_3_O_4_ cubes and Co_3_O_4_ nanosheets-based electrodes in 2 M KOH solution with the fitted equivalent circuit in the inset

The ion migration and charge transfer characteristics of the Co_3_O_4_ nanocubes and nanosheets-modified electrodes were further investigated by EIS measurement and the results were shown in Fig. 8. As we can see that a semicircle in high-frequency region and a straight line in low-frequency region appear in the corresponding Nyquist plot. The internal resistance (*R*_s_) refers to the sum of the ionic internal resistance of electrolyte, the internal resistance of active material, and the contact resistance between electrode material and electrolyte. The *R*_s_ value is reflected by the intercept of the semicircle on the real axis (*Z*’). The resistance of charge transfer reflected by the diameter of the semicircle, the smaller of the diameter, the better transfer of the ions between electrolyte and active material. The Warburg impedance (*Z*_W_) can be reflected by the slope of the straight line in low frequency, and *Z*_W_ is mainly caused by the diffusion of OH^−^ ions in electrolyte. In the inset of Fig. 8 is the equivalent circuit fitted from the EIS data, from which a better understanding can be obtained. By analyzing the EIS results of the two electrodes, the *R*_s_ were found to be 0.78 and 0.72 Ω for Co_3_O_4_ nanocubes and nanosheets-modified electrodes, respectively, which may be attributed to the fact that the thinner sheet-like structure is more favorable for ion permeation in the electrolyte than the cubic structure. Furthermore, the *R*_ct_ value of the two kind of electrodes were 6.9 and 4.1 Ω, respectively, suggesting that the nanosheets-modified electrode provided higher charge transfer capability.

## Conclusions

Porous Co_3_O_4_ quasi-cubes were prepared through an egg albumin-assisted hydrothermal method with a subsequent high-temperature treatment of precursor in air directly. The size and shape of final Co_3_O_4_ samples had a close relationship with the amount of egg albumin and hydrothermal reaction time, respectively. Such Co_3_O_4_ cubes possessed a mesoporous characteristic with surface area of 80.3 m^2^/g, average pore size of 5.58 nm, and main pore size distribution at 4.03 nm. Once these Co_3_O_4_ quasi-cubes were processed into a working electrode, it delivered a high specific capacitance of 754 F g^−1^ at 1 A g^−1^ and 581 F g^−1^ at the current density of 10 A g^−1^. After a continuous 4000 cycles at 5 A g^−1^, 86.7% capacitance retention could be obtained and it demonstrated a good cycling stability. The outstanding electrochemical properties of these Co_3_O_4_ cubes enable them to be promising electrode materials for advanced supercapacitors. In addition, the egg albumin-assisted synthesis route is expected to be extended to prepare other oxides-based electrode materials with novel morphology and superior electrochemical performances.

## Data Availability

The datasets used and/or analyzed during the current study are obtained from the corresponding author on reasonable request.

## Abbreviations

BET: Brunauer-Emmett-Teller
CNTs: Carbon nanotubes
CP: Chronopotentiometry
C_s_: Specific capacitance
CV: Cyclic voltammetry
EDLCs: Electric double-layer capacitors
EIS: Electrochemical impedance spectroscopy
FESEM: Field-emission electron microscope
NMP: N-methyl-2-pyrrolidone
NP: Nanoparticle
PCs: Pseudo-capacitors
PVDF: Polyvinylidene fluoride
R_s_: Internal resistance
SAED: Selected area electron diffraction
SCE: Saturated calomel electrode
TEM: Transmission electron microscope
TMOs: Transition metal oxides
XPS: X-ray photoelectron spectroscopy
XRD: X-ray diffraction
Z_W_: Warburg impedance
